# SH-1028, An Irreversible Third-Generation EGFR TKI, Overcomes T790M-Mediated Resistance in Non-Small Cell Lung Cancer

**DOI:** 10.3389/fphar.2021.665253

**Published:** 2021-04-27

**Authors:** Luwei Han, Xiaomeng Zhang, Zhiqiang Wang, Xian Zhang, Liwen Zhao, Wei Fu, Xiaobo Liang, Zhibo Zhang, Yong Wang

**Affiliations:** ^1^ Department of Pharmacy, China Pharmaceutical University, Nanjing, China; ^2^ Nanjing Sanhome Pharmaceutical Co., Ltd., Nanjing, China; ^3^ Laboratory of Drug Screening, China Pharmaceutical University, Nanjing, China

**Keywords:** SH-1028, osimertinib, EGFR-T790M, non-small cell lung cancer, pharmacodynamics

## Abstract

SH-1028 is an irreversible third-generation EGFR TKI. Both SH-1028 and osimertinib have a pyrimidine structure (a typical mutant-selective EGFR TKI structure). Compared with osimertinib, SH-1028 is modified on the indole ring, thus resulting in a more stable 6,7,8,9-tetrahydro-pyrrolo [1, 2-a] indol structure. In this study, we explored the anti-tumor effect of SH-1028 *in vitro* and *in vivo*, the inhibition of cell signal, such as EGFR and ERK phosphorylation, and verified the relationship between the pharmacokinetics and pharmacodynamic responses. Firstly, SH-1028 selectively inhibited EGFR sensitive and resistant mutations, with up to 198-fold more effective compared with wild-type EGFR cells. Then, in mouse xenograft models, oral administration of SH-1028 at a daily dose of 5 mg/kg significantly inhibited proliferation of tumor cells with EGFR sensitive mutation (exon 19 del) and resistant mutation (T790 M) for consecutive 14 days, with no TKI-induced weight loss. Moreover, SH-1028 exhibited good bioavailability, and was distributed extensively from the plasma to the tissues. The main metabolite of SH-1028, Imp3, was tested and showed no wild-type EGFR inhibition or off-target effects. In conclusion, SH-1028 is a new third-generation EGFR inhibitor that exhibits potent activity against EGFR sensitive and resistant (T790 M) mutations.

## Introduction

Lung cancer is the leading cause of cancer death among both men and women (Paez et al., 2004; Siegel et al., 2020). More than half of patients with lung cancer die within one year of diagnosis and the 5-years survival rate is approximately 17.8% (Zappa and Mousa, 2016). Non-small cell lung cancer (NSCLC) accounts for ∼85% of all lung cancers. Gene mutation has been recognized to play critical roles in the progression and metastasis of NSCLC, including the mutations in *EGFR* and *ALK* genes (Herbst et al., 2008).

Epidermal growth factor receptor (EGFR) is a receptor protein that penetrates the cell membrane and interacts with other signaling molecules. The kinase domain consists of N- and C-terminal lobes, and an adenosine triphosphate (ATP) is bound in the cleft between the two lobes. Generally, after a ligand binds to a receptor, an asymmetric dimer is formed and the phosphate group from ATP is transferred to the tyrosine residue of the regulatory domain. When the EGFR extracellular domain binds to its ligands, such as epidermal growth factor (EGF) and transforming growth factor-α (TGF-α), it forms dimers with other EGFR or other HER family members and undergoes autophosphorylation at the key tyrosine residues, thus activating several downstream signaling pathways such as protein kinase B (AKT/PKB) and mitogen-activated protein kinases (MAPK). These pathways can regulate multiple cellular processes, including proliferation, survival and apoptosis (Normanno et al., 2006; Gazdar, 2009). The constitutive activation of EGFR signaling, caused by gene mutations, gene amplification or both, has been demonstrated to be closely associated with the initiation, progression and poor prognosis of NSCLC. The two most common EGFR active mutations are small in-frame deletions in exon 19 (particularly E746-A750del) and amino acid substitution in exon 21 (L858R), which collectively account for >90% of known activating EGFR mutations (44.8 and 39.8%, respectively) (Paez et al., 2004; Gazdar, 2009; Cheng et al., 2015). Thus, the mutations in *EGFR* gene can serve as both predictive biomarkers and drug targets for targeted therapy.

Gefitinib, erlotinib and icotinib, as the first-generation EGFR tyrosine kinase inhibitors (TKIs), greatly improve the prognosis of NSCLC patients with *EGFR* gene mutations (Ke and Wu, 2016; Goss et al., 2016; Lee, 2017). Although EGFR TKIs can exert an excellent therapeutic effect on EGFR mutation-positive NSCLC, most patients develop resistance to these agents after 9–13 months. As reported previously, more than 50% of patients who developed resistance to EGFR TKIs harbor the T790M mutation (Kobayashi et al., 2005; Fukuoka et al., 2011; Yu et al., 2013). Afatinib, the second-generation EGFR TKI, has been demonstrated to inhibit EGFR T790M-resistant mutation irreversibly in animal models and NSCLC patients, but its poor selectivity toward wild-type EGFR could result in severe side effects at the clinical stage (Edwards et al., 2018). In 2015, FDA granted accelerated approval to osimertinib, the third-generation EGFR TKI that irreversibly binds to EGFR kinase by targeting the cysteine residue at codon 797 (C797). This agent is highly selective against T790M mutation over wild-type EGFR, and can be used to treat T790M-mutated NSCLC patients. In clinical, osimertinib is given as a second-line treatment to the locally advanced or metastatic (stage IIIB/IV) NSCLC patients who have progressed following prior EGFR TKI therapy, and provides good clinical activity with minimal side effects (ORR = 70%) (Goss et al., 2016; Mok et al., 2017). In addition, osimertinib demonstrates superior efficacy compared to the first-line EGFR TKIs in advanced NSCLC patients with EGFR mutations (18.9 vs. 10.2 months) and prolongs the overall survival significantly (38.6 months in osimertinib group vs. 31.8 months in the comparator group), with a similar safety profile and lower rates of serious adverse events (Soria et al., 2018; Ramalingam et al., 2020).

Apart from osimertinib, several third-generation EGFR TKIs are currently being developed in China, such as AC0010, AST2818 and HS-10296, through phase I-III clinical trials (Xu et al., 2016; Yang et al., 2020; Shi et al., 2020). SH-1028 is a pyrimidine-based irreversible EGFR inhibitor, which selectively and specifically inhibits EGFR active mutations and T790M resistance mutation, while sparing wild-type EGFR. In the preclinical studies, SH-1028 exhibited potent inhibition against EGFR TKI-sensitive mutation and EGFR T790M mutation *in vitro* and *in vivo*. Importantly, SH-1028 and its metabolite (Imp3) can result in greater selectivity for wild-type EGFR, which is distinct from AZ5104 (a major metabolite of osimertinib).

In this study, we explored the anti-tumer effect of SH-1028 in cell lines and xenograft models, the inhibition of cell signal, such as EGFR and ERK phosphorylation, and verified the relationship between the pharmacokinetics and pharmacodynamic responses. It is therefore warranted to further develop SH-1028 as an alternative therapeutic agent for NSCLC patients who acquired resistance to first-generation EGFR TKIs or those with EGFR mutation-positive advanced NSCLC.

## Materials and Methods

### Cell Lines, Animals and Reagents

Human lung cancer cell lines (NCI-H1975, H3255 and A431) were obtained from the American Type Culture Collection (ATCC), while PC-9 cell line was supplied by the European Collection of Authenticated Cell Cultures (ECACC). For *in vivo* implantation, NCI-H1975, H3255 and PC-9 cells were cultured in RPMI1640 (Invitrogen) supplemented with 10% v/v fetal calf serum (Sigma-Aldrich) and 2 mmol/L *l*-glutamine (Invitrogen). A431 cells were cultured in DMEM (Invitrogen) supplemented with 10% v/v fetal calf serum and 2 mmol/L v/v *l*-glutamine. All cell lines were then cultured in a humidified incubator with 5% CO_2_ at 37°C.

The female Nu/Nu nude mice were purchased from Beijing Vital River Laboratories. All experiments involving animal handling, care, and treatment were carried out in Beijing pharmaron Co., Ltd. according to the guidelines and SOPs approved by the Department of Science and Technology of Beijing Province, China (No. SYXK 2012-0018). Ethical approvals for the mice experiments were obtained from the Beijing Pharmaron Co., Ltd. The experimental procedures were in compliance with the Association for Assessment and Accreditation of Laboratory Animal Care international (#001322).

### Molecular Docking

Molecular modeling/docking structures were conducted using Autodock, MGL Tools, and Pymol. The crystal structure of T790M-mutant EGFR in complex with osimertinib was retrieved from the protein data bank (PDB entry 6JX0). Docking was performed with the Autodock program. The MGL Tools was used to prepare the protein. During protein preparation, conserved water molecules within 10 Å distance of osimertinib were not deleted. For docking, the scoring grids were centered on the crystal structure of osimertinib using the default bounding box sizes, with an inner box of 10 Å on each side and an outer box of 24 Å on each side. Covalent docking with default parameters was used. The best docked poses were selected as the ones with the lowest docking score.

### Kinase Inhibition Assay

The inhibitory activities of test compounds against human EGFR kinases (wild type, L858R, d746-750, d746-750/T790M, T790M/L858R and L861Q) were determined using homogenous time-resolved fluorescence (HTRF) assay. The enzyme reaction contains recombinant N-terminal GST-tagged human EGFR (T790M/L858R) that phosphorylates the HTRF biotinylated peptide substrate. The sequence of the substrate is proprietary to CisBio. All compounds were serially diluted in 100% (v/v) DMSO before being acoustically dispensed from an Echo 555 (Labcyte) into black Corning 1536-well assay plates (From 10 to 0.001 μmol/L for wild type kinase and From 1 to 0.0001 μmol/L for other kinase). Kinase activity assays were performed in a total reaction volume of 3 μl per well. The enzyme mixture (1.5 μl) consisted of 1.6 nmol/L EGFR (T970M, L858R), 1 mmol/L DTT, and 10 mmol/L MgCl_2_. The substrate mixture (1.5 μl) consisted of 1 μmol/L TK substrate, 30 μmol/L ATP, 1 mmol/L DTT, and 10 mmol/L MgCl_2_. Following a 50-min incubation, 3 μl of stopping mixture was added, which consisted of 250 nmol/L Strep-XL665 and TK Ab-Cryptate diluted in kit detection buffer. The plates were incubated for 1 h before being read on Pherastar using standard HTRF settings. N-terminal GST-tagged recombinant human EGF receptor, with amino acids 696-end containing the mutations T790M and L858R, was obtained from Millipore. There was no pre-incubation of the test compounds with the enzyme/substrate mixture prior to ATP addition. For IC50 calculation, data were analyzed using XLFit version 5.3.

### Cell Proliferation Assay

Cells in the exponential growth phase were collected and counted to seed in 96-well plates at a density of 500–2000 cells per well and incubated at 37°C for 24 h. Test compounds were dissolved in DMSO as a 10 mmol/L stock solution. The initial concentration of compounds were 10 μmol/L and the 3-fold dilution was nine times. The cells added with test compounds were placed in a 37°C incubator for 72 h. After 72 h of treatment, 50 µl CTG solution that had been melted and equilibrated to room temperature was added in each well. Plate was shaked for 2 min and the mixture was incubated at room temperature for 10 min. Then the luminescent signal was valued using Envision 2104 plate reader and calculated as follows:

$$\text{\%Inhibition}=100\text{\%}\times \;\frac{{\text{Lum}}_{\text{Vehicle}}-{\text{Lum}}_{\text{Sample}}}{{\text{Lum}}_{\text{Vehicle}}-{\text{Lum}}_{\text{NC}}}$$

Lum_Vehicle_, the luminescent signal of DMSO treatment group; Lum_Sample_, the luminescent signal of test compound treatment group; Lum_NC_, the luminescent signal of culture medium.

The half-maximal inhibition concentration (IC_50_) values were processed by GraphPad Prism7 (GraphPad Software; La Jolla, CA).

### Phosphorylation of EGFR Assay

Cells (NCI-H1975/PC-9/A341) were seeded into a 384-well plate (10,000 cells/well), grown for 24 h, and then treated with different test compounds (0.1 μmol/L) for 1, 2, 4, 6, 8 and 10 h. A431 cells were stimulated with 100 ng/ml EGF after incubation for 10 min. Cells were washed with ice-cold HBSS before extraction with 25 μl cell lysis buffer. Phosphorylation of EGFR was measured using a sandwich ELISA assay with phospho-specific EGFR (pY1068) antibodies [AlphaLISA SureFire *p*-EGF Receptor (Tyr1068) Assay Kit, PerkinElmer].

Cells (NCI-H1975/PC-9/A341) were seeded into a 384-well plate (10,000 cells/well), grown for 24 h, and then treated with 0.1 μmol/L of test compounds for 2 h. Cells were washed with fresh medium once. Then, 50 μl of medium was added in each well and the plate was cultured in an incubator for 0, 1, 2, 6 and 24 h. Cells were washed with ice-cold Hanks Balanced Salt Solution (HBSS) before extraction with 25 μl cell lysis buffer. The phosphorylation levels of EGFR were measured as described above.

### Pharmacodynamic Analysis *In Vivo*

Nu/Nu nude mice were purchased from the Beijing Vital River Laboratories of China. The 6–8 weeks old female mice were inoculated subcutaneously at the right flank with approximately 3–5 × 10^6^ tumor cells in 0.2 ml of medium. The treatments were started when the tumor size reached approximately 200 mm^3^. For NCI-H1975 and A431 models, mice were divided into five groups (*n* = 6 per group) including a vehicle group (citric acid-sodium citrate buffer solution, pH 2.75), three SH-1028 treatment groups (2.5, 5, and 15 mg/kg), and a control group (osimertinib, 5 mg/kg). All mice were orally administrated once daily for consecutive 14 days. Mouse body weight, tumor weight and tumor volume (TV) were measured twice per week. TV was then used for the calculation of tumor inhibitory rate and tumor regression rate.

Paraffin-embedded tumor tissues from each group were sectioned, mounted on positively charged glass slides, baked, deparaffinized, and rehydrated. Antigen retrieval was performed by heating slides in EDTA (pH 8.0) for 10 min. Sections were incubated in *p*-EGF Receptor (Y1868) Rabbit antibody and Phospho-p44/42 MAPK (Erk1/2) (Thr202/Tyr204) (20G11) Rabbit antibody overnight at 4°C and then incubated with Anti-Rabbit IgG (H + L) at 37°C for 45 min. The sections were then stained with diaminobenzidine (DAB) and visualized.

### Pharmacokinetics Analysis *In Vivo*

NCI-H1975 tumor-bearing mice with tumor burden of approximately 200 mm^3^ were used in the pharmacokinetics and pharmacodynamics study. The mice were administered with 2.5, 5 or 15 mg/kg of SH-1028 on a daily basis for 14 consecutive days. Blood samples were collected at 0 (pre-dose), 10, 30 min, 1, 2, 4, 8, and 24 h post-dose. Tumor tissues, lung and blood samples from the 15 mg/kg SH-1028 treatment group were collected at 0.5, 1, and 2 h post-dose on Day 1. The plasma and tissue concentrations of SH-1028 (m/z 540.40 to 72.10) and its metabolite Imp3 (m/z 526.50 to 469.40) were analyzed by the LC/MS/MS (AB Sciex API 5500 Qtrap) system in positive

Ionization mode. Drug to internal standard peak area ratios for the standards were used to create a calibration curve using 1/×^2^ weighted least-squares regression analysis. Concentrations of each analyte were quantified by comparing the ratios for each in-trial sample with those in the relevant calibration curve.

Metabolite profiling was conducted in mouse, rat, dog, monkey, and human liver microsomes *in vitro* as well as in rat plasma, feces, and urine samples *in vivo* (From other experiments). SH-1028 was incubated with liver microsomes for 60 min at 37°C. All samples were analyzed by the LC/MS/MS (AB Sciex, API 5500 Qtrap) system. The structures of major metabolites were elucidated based on EPI spectra and further verified using authentic standards. The main metabolite Imp3 was quantified in multiple reaction monitoring mode in rat, dog and human clinical samples. The biological activities of SH-1028 and its major metabolites against wild-type and mutant EGFR were assessed using cell proliferation assay.

### Statistics Analysis

Kinase inhibition assay, cell proliferation assay and phosphorylation assay of EGFR underwent technically repeats. Pharmacokinetics and pharmacodynamic analysis *in vivo* underwent biological repeats. Data were expressed as “mean ± SD” and two-sample *t* test was used to compare the statistical differences between two independent counting samples. All data were analyzed using Graphpad Prism 7.0 and two-sided *p* < 0.05 was considered a significant difference.

## Results

### SH-1028 is a Mutant-Selective Inhibitor of EGFR Kinase Activity

**FIGURE 1:**
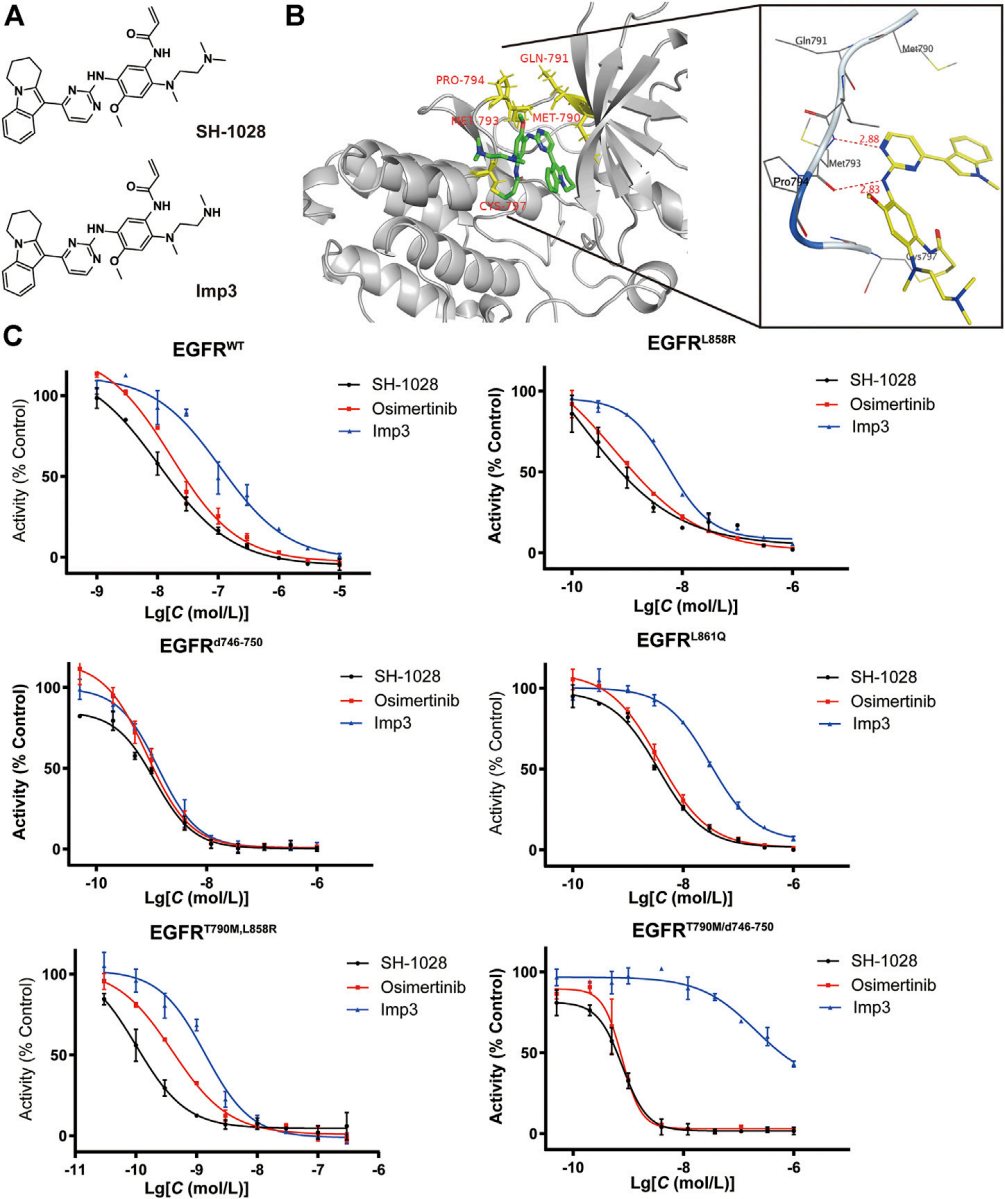
Preliminary efficacy of SH-1028. (**A**) Chemical structure of SH1028 and Imp3. (**B**) Docking structure of SH1028 to T790M EGFR. (**C**) kinase inhibition assay of SH-1028 in EGFR (WT, L858R, d746-750, L861Q, T790M/L858R, T90M/d746-750). Data were expressed as mean ± SD (*n* = 3).

SH-1028 was an irreversible third-generation EGFR TKI (Figure 1A), and molecular docking indicated that it could bind irreversibly to EGFR kinase by targeting cysteine-797 residue in the ATP binding site via covalent bond formation (Figure 1B). Firstly, in EGFR kinase enzymatic assay (Table 1; Figure 1C), SH-1028 showed apparent inhibitory effects on EGFR^L858R^, EGFR^L861Q^, EGFR^L858R/T790M^, EGFR^d746–750^ and EGFR^d746–750/T790M^ kinases, with IC_50_ values of 2.35, 13, 0.55, 1.6 and 0.84 nmol/L, respectively. The drug exhibited nearly 80 times greater potency against L858R/T790 M than wild-type EGFR, which was consistent with the design goal of a mutant selective EGFR TKI. Compared with osimertinib, SH-1028 demonstrated a stronger inhibitory effect on EGFR^L858R^ and similar inhibitory effects on EGFR^L858R/T790M^, EGFR^d746–750^ and EGFR^d746–750/T790M^. In addition to that, SH-1028 exhibited higher selectivity toward EGFR^WT^ than osimertinib. Subsequent *in vivo* studies revealed that SH-1028 was metabolized to produce a primary metabolite, Imp3 (Figure 1A). The inhibitory activities of Imp3 against EGFR^L858R^, EGFR^L861Q^, EGFR^L858R/T790M^, EGFR^d746–750^ and EGFR^d746–750/T790M^ kinases were slightly weaker than those of SH-1028, with IC_50_ values of 8, 36.5, 2, 2.3 and 1.1 nmol/L, respectively. However, it is worth noting that Imp3 maintains a good selectivity for wild-type EGFR kinase.

**TABLE 1:** Kinase enzymatic assay ($\bar{X}$; *n* = 3).

Kinase type	IC_50_ (nmol/L)		
	SH-1028	Imp3	Osimertinib
EGFR^WT^	18	144	30
EGFR^L858R^	0.7	6	1
EGFR^d746–750^	1.4	1.7	0.93
EGFR^L861Q^	4	32	4
EGFR^L858R/T790M^	0.1	1	0.1
EGFR^d746–750/T790M^	0.89	1.1	0.9

Then, to explore a broader kinase selectivity profile, SH-1028 and its metabolite Imp3 at 1 μmol/L were tested over 371 human kinases available on a commercial biochemical kinase panel (Eurofins). SH-1028 showed minimal off-target kinase activity, with only a limited number of additional kinases had more than 60% inhibition at 1 μmol/L and moderate IC_50_ potencies, including ACK1, ALK, BLK, ErbB2(h), ErbB4(h) and MNK2 (Table 2). In addition, SH-1028 and Imp3 also exerted weak inhibitory effects against insulin-like growth factor 1 receptor (IGF1R) and insulin receptor (IR). These receptors have a methionine gatekeeper in their kinase domains, and are related to hyperglycemia in other clinical trials of EGFR TKI, such as Co-1686.

**TABLE 2:** Kinase panel ($\bar{X}$; *n* = 2).

kinase	SH-1028 %kinase inhibition (μmol/L)	SH-1028 %IC_50_ kinase inhibition (nmol/L)	Imp3 %kinase inhibition (μmol/L)	Imp3 %IC_50_ kinase inhibition (nmol/L)
ACK1(h)	81	86	90	124
ALK(h)	85	79	92	53
Blk(h)	83	143	85	229
BRK(h)	85	62	91	80
ErbB2(h)	72	55	83	197
ErbB4(h)	90	46	92	84
FAK(h)	68	235	87	267
Fer(h)	45	542	80	452
Fes(h)	46	557	74	309
Flt3 (D835Y) (h)	86	78	99	41
Flt3(h)	88	71	97	45
Flt4(h)	92	62	96	104
HIPK4(h)	67	232	82	276
IGF-1R(h)	38	574	70	812
IGF-1R(h), activated	46	499	64	709
IR(h), activated	58	241	74	321
Itk(h)	66	320	68	825
JAK3(h)	61	1750	55	1,374
LOK(h)	24	1949	72	593
LRRK2(h)	89	82	93	120
LTK(h)	83	81	93	61
Met(h)	46	737	73	486
MLCK(h)	55	400	68	597
Mnk2(h)	63	23	69	22
MYO3B(h)	58	349	67	506
PTK5(h)	39	644	64	736
Pyk2(h)	73	228	83	227
Ros(h)	96	50	98	47
Tec(h) activated	80	150	77	272
TSSK1(h)	84	104	98	73

### SH-1028 Potently and Selectively Targets Mutant EGFR Cell Lines *In Vitro*

**FIGURE 2:**
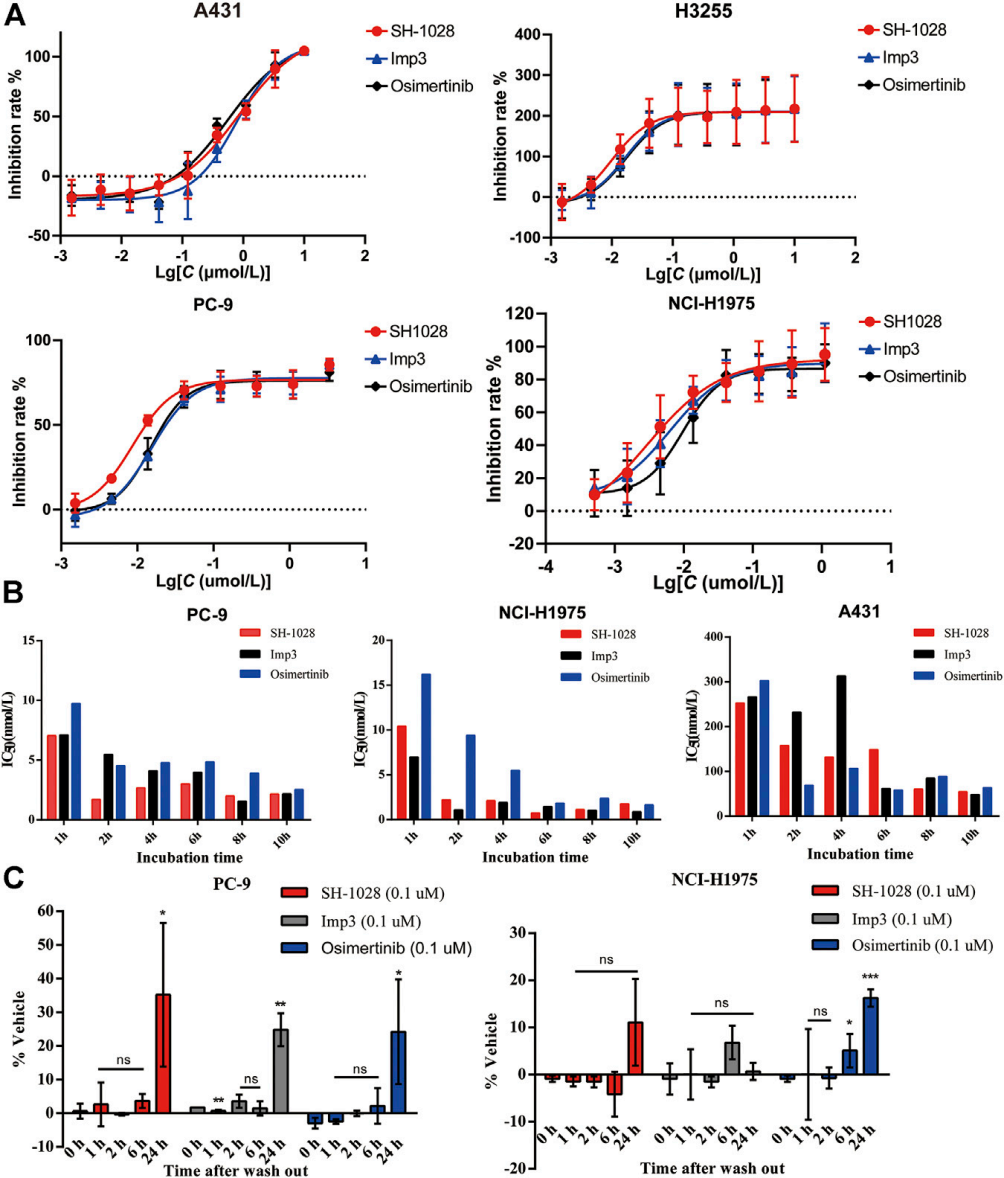
The efficacy of SH-1028 in cell lines. (**A**) The antiproliferative capabilities of SH-1028 to A431 (EGFR^WT^), H3255 (EGFR^L858R^), PC-9 (EGFR^d746–750^) and NCI-H1975 (EGFR^L858R/T790M^). (**B**) Time-dependent inhibition of EGFR phosphorylation in PC-9, NCI-H1975 and A431. (**C**) Inhibition of EGFR phosphorylation. after the drug wash out in PC-9 and NCI-H1975. Data were expressed as mean (*n* = 2; **B**) and mean ± SD (*n* = 3; **A** and **C**); *p < 0.05 vs. 0 h; **p < 0.01 vs. 0 h; ***p < 0.001 vs. 0 h; ns: no significant difference vs. 0 h.

The antiproliferative capabilities of SH-1028 and Imp3 were compared with osimertinib using a number of tumor cell lines harboring either the wild-type or mutant forms of EGFR. SH-1028 selectively inhibited EGFR-mutated NCI-H1975, H3255 and PC-9 cells, with IC50 values of 3.93, 9.39 and 7.63 nmol/L, respectively, which were about 198-, 83- and 102-fold more sensitive than the inhibition of wild-type EGFR in A431 cells (Figure 2A; Table 3). Compared with osimertinib, SH-1028 showed an improved inhibitory effect on the proliferation of EGFR sensitive or resistance mutant cells. Besides, the inhibitory effect of SH-1028 against A431 (EGFR wild-type) cells was slightly weaker than that of osimertinib, indicating that SH-1028 is a selective inhibitor of EGFR mutant cells. As opposed to osimertinib and its metabolite AZ5104, Imp3 (the primary metabolite of SH-1028) also exerted a weak inhibitory effect on EGFR wile-type cell line (Figure 2A; Table 3). This indicates that even though SH-1028 is metabolized to Imp3, it still has a greater selectivity for mutant EGFR compared with wild-type EGFR.

**TABLE 3:** The IC_50_ of compounds to cell lines ($\bar{X}\pm S$; *n* = 3).

Cell	IC_50_ (nmol/L)		
	SH-1028	Imp3	Osimertinib
A431 (EGFR^WT^)	778.89 ± 134.74	704.09 ± 92.50	466.47 ± 86.18
H3255 (EGFR^L858R^)	9.39 ± 0.88	17.15 ± 0.55	17.78 ± 0.50
PC-9 (EGFR^d746–750^)	7.63 ± 0.18	15.02 ± 0.65	14.17 ± 1.63
NCI-H1975 (EGFR^L858R/T790M^)	3.93 ± 1.12	6.56 ± 1.43	10.89 ± 1.42

### SH-1028 Inhibits EGFR Phosphorylation in a Time-Dependent Manner *In Vitro* and Maintains the Inhibitory Activity After Wash-Out

The phosphorylation levels of EGFR in PC-9, NCI-H1975 and A431 lines were determined by immunoblot analysis (Figure 2B). SH-1028, Imp3 and osimertinib remarkably decreased the phosphorylation levels of EGFR with prolonged incubation time, and displayed a significant time dependence in all cell lines. Similar to the results of cell proliferation study, the inhibitory effect of SH-1028 on EGFR phosphorylation in PC-9 cells was stronger than that of osimertinib, while the inhibitory effects of Imp3 on EGFR phosphorylation in tumor cell lines were relatively similar to those of osimertinib at various time points. In NCI-H1975 cells, SH-1028 and Imp3 exerted stronger inhibitory effects on EGFR phosphorylation compared to osimertinib, indicating that SH-1028 has strong inhibitory activity against EGFR mutant kinases *in vitro*. However, in A431 cells, SH-1028 displayed a weak inhibitory effect on EGFR phosphorylation, which was consistent with the above selectivity results.

To confirm the irreversible binding mechanism of SH-1028, the cells were incubated with 0.1 μmol/L of SH-1028 and Imp3 for 2 h, and subsequently washed out. The phosphorylation levels of EGFR in NCI-H1975 and PC-9 cells showed no significant increases within 6 h. After 24 h of wash-out, the phosphorylation levels of EGFR in these cells were recovered (Figure 2C). This suggests that SH-1028 and Imp3 continuously inhibit the phosphorylation of EGFR in PC-9 and NCI-H1975 cells at lower concentrations or even drug-free for at least 6 h. Furthermore, the duration and degree of inhibition were similar to those of osimertinib.

### SH-1028 Inhibits EGFR-Mutant Tumor Progression but Not Wild-Type EGFR *In Vivo*

**FIGURE 3:**
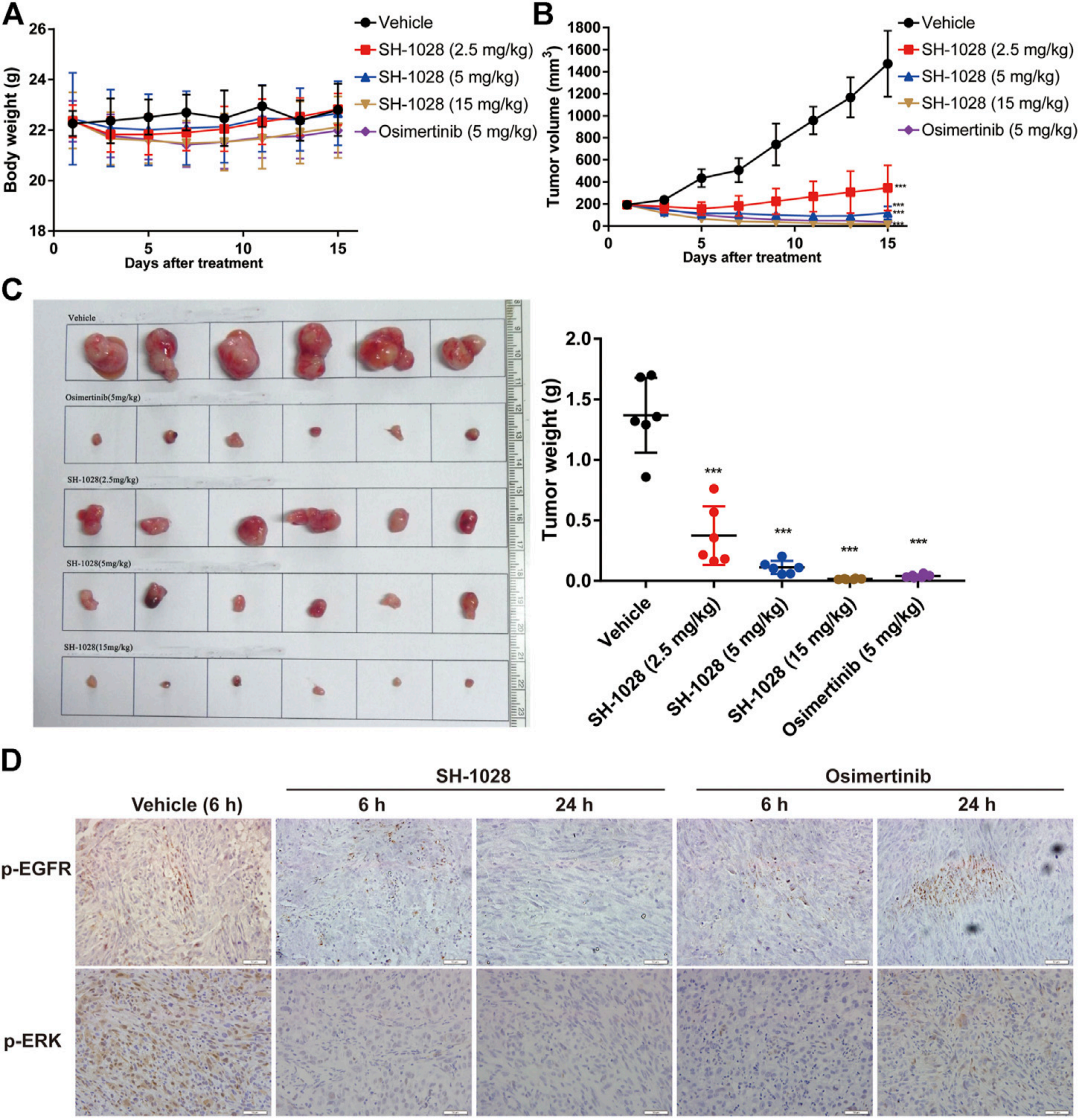
The efficact of SH-1028 *in vivo.* (**A**) The body weight of NCI-H1975 xenograft models after treatment (*n* = 6). (**B**) The tumor growth curve of NCI-H1975 xenograft models after treatment (*n* = 6). (**C**) The tissue and weight of tumor from NCI-H1975 xenograft models (*n* = 6). (**D**) The immunohistochemistry of tumor tissue from NCI-H1975 xenograft models. Data were expressed as mean ± SD; ***p < 0.001 vs. Vehicle group (Day 15).

To evaluate the anti-tumor effect of SH-1028 *in vivo*, different xenograft models were established using human lung cancer cell lines. Oral administration of SH-1028, ranging from 2.5 to 15 mg/kg, could lead to a significant inhibition of tumor cell growth in both PC-9 (exon 19 del) and NCI-H1975 (L858R/T790M) xenograft models (Supplementary Figure S1A; Figure 3B). The results also demonstrated that SH-1028 induced profound shrinkage at low doses against both EGFR TKI-sensitizing and T790M + resistant tumor xenograft models. Compared with the vehicle control mice, the tumor growth inhibition rates of 2.5, 5 and 15 mg/kg SH-1028 treatment groups were 72.6, 91.8, and 98.8%, respectively, after 14 days of dosing in NCI-H1975 model (Figure 3C). Moreover, no TKI-induced weight loss was observed (Figure 3A). Besides, the comparative efficacy of SH-1028 against wild-type EGFR was evaluated using A431 xenograft model (Supplementary Figure S1B). After treatment with 5 mg/kg/day SH-1028, a moderate tumor growth inhibition was observed in A431 (wild-type EGFR) tumor xenografts, suggesting that SH-1028 and its metabolites are not entirely inactive against tumor cells with wild-type EGFR. Similarly, the same 5 mg/kg/day dose level of SH-1028 was sufficient to induce profound and sustained tumor shrinkage in both NCI-H1975 and PC-9 xenograft models with EGFR mutations. It is noteworthy that afatinib, the second-line EGFR TKI, exhibits a significant inhibition on tumor growth in A431 xenograft model at the dose of 30 mg/kg (a clinically representative dose) (Supplementary Figure S3B), and the mice demonstrated severe body loss due to a poor selectivity of wild-type EGFR (Supplementary Figure S1C; Gurtner et al., 2014).

Meanwhile, the phosphorylation levels of EGFR and ERK in tumor tissues of the NCI-H1975 xenograft model were analyzed by immunohistochemistry (Figure 3D). The results showed that SH1028 could continuously inhibit the phosphorylation of EGFR and ERK, maintaining the expression at extremely low level in 6–24 h. However, at 24 h after osimertinib treatment, the expression of phosphorylated protein was slightly higher than the SH1028 treatment group, which confirmed that SH1028 had a longer-lasting inhibitory effect.

### SH-1028 Shows Good Bioavailability, and is Distributed Extensively From the Plasma to the Tissues

**FIGURE 4:**
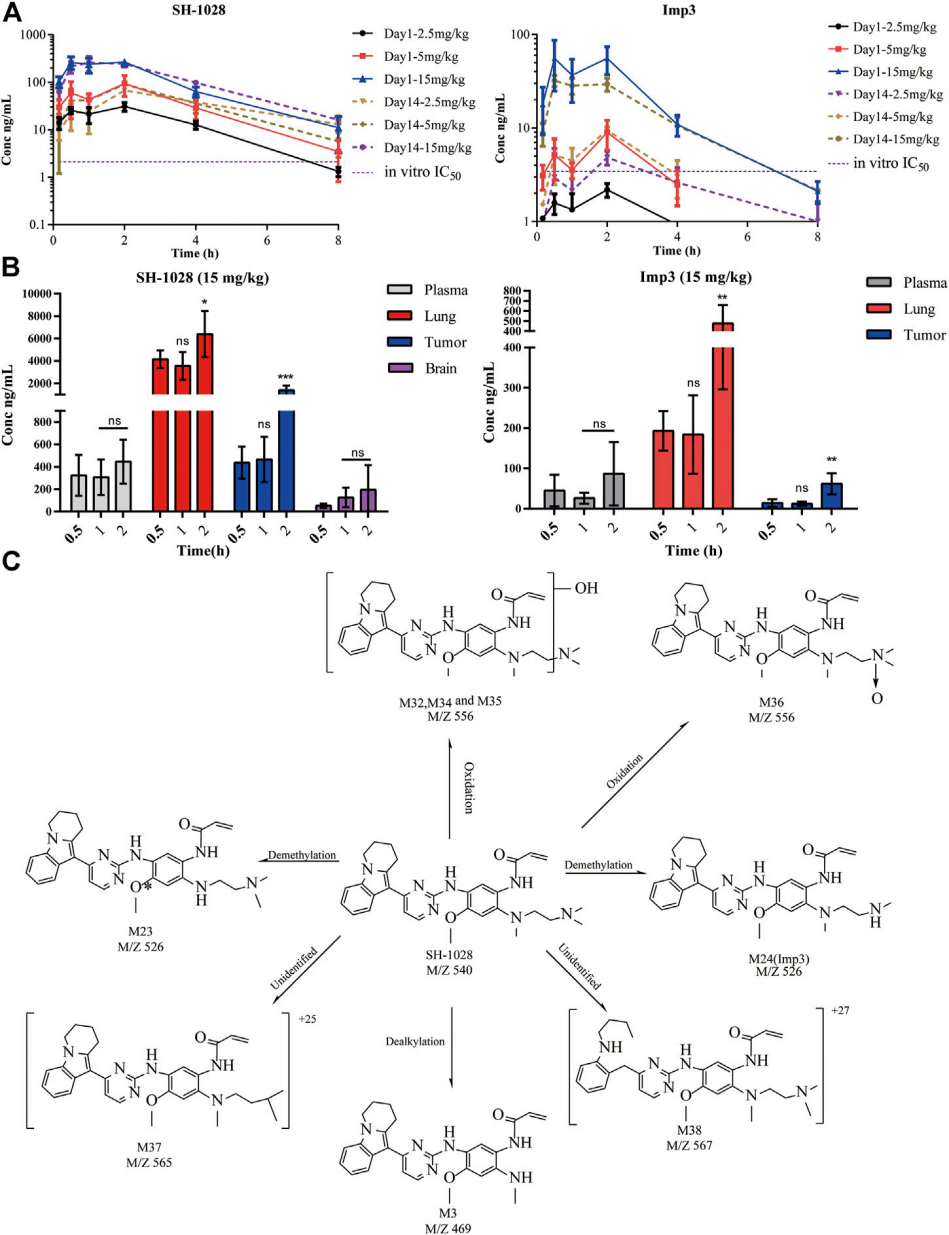
The pharmacokinetic characteristics of SH-1028. (**A**) SH-1028 and Imp3 concentrations in plasma from SH-1028-treated mice on days 1 and 14; p. o.:oral administration (*n* = 6). (**B**) SH-1028 and Imp3 concentrations in tissues from SH-1028-treated mice on days 1 (*n* = 6). (**C**) Proposed SH-1028 metabolic pathways and its major metabolites. Data were expressed as mean ± SD. *p < 0.05 vs. 0.5 h; **p < 0.01 vs. 0.5 h; ***p < 0.001 vs. 0.5 h; ns: no significant difference vs. 0.5 h.

To explore the pharmacokinetics/pharmacodynamics relationship *in vivo*, the plasma and tumor tissues of NCI-H1975 tumor-bearing mice were examined after oral administration of 2.5, 5, and 15 mg/kg SH-1028 for 1 day or 14 consecutive days. The results showed that SH-1028 was absorbed with the T_max_ of 1.5–2 h, indicating SH-1028 is rapidly distributed into tissues, including lung tumor tissues. The AUC_0–t_ values of SH-1028 in plasma were 118, 300 and 931 ng × h/ml on Day 1, while 272, 308 and 993 ng × h/ml on Day 14, respectively. Moreover, there was no significant accumulation of SH-1028 between Days 1–14 (Figure 4A). SH-1028 was rapidly metabolized to Imp3 *in vivo*, and the total exposure level (AUC) was approximately 18.8% compared with the parent compound (Figure 4B). Besides, 15 mg/kg of SH-1028 was rapidly and extensively distributed into tissues within 2 h, including tumor tissues, adjacent lung tissues and brain in a time dependent manner. (Figure 4C). In NCI-H1975 tumor-bearing mice, Imp3 could be detected in plasma and tissues within 0.5 h after dosing (Figure 4D). These results indicated that SH-1028 was first metabolized to Imp3 and then distributed to other tissues.

### SH-1028 Is Metabolized to Several Metabolites Through Phase I Reactions

In this study, 27 metabolites of SH-1028 were detected in mouse, rat, dog, monkey and human liver microsomes. SH-1028 was mainly eliminated through phase I metabolic reactions, such as demethylation, oxidation and dealkylation, in the liver microsomes. Figure 4E displayed the major metabolites of SH-1028, and the corresponding metabolic pathways were N-demethylation (M23 and M24), N-oxidation (M36) and N-dealkylation (M3). All metabolites were detected in all species, and no human-specific metabolite was found in the liver microsomes. One of the main metabolite was Imp3, which had been identified and analyzed earlier.

## Discussion

Lung cancers is generally classified into two main types: NSCLC and small cell lung cancers (SCLC). NSCLC accounts for 85%, while SCLC accounts for 15% of all lung cancers. In China, the death rate of lung cancer accounts for 22.7% of all malignant tumors, and the incidence rate is increasing year by year (Chen et al., 2016; Cao and Chen, 2019; Yang D. et al., 2020). It is expected that the number of lung cancer patients in China will reach 1 million in 2025. Among East Asian patients with NSCLC, *EGFR* mutations are the most common, accounting for about 22.2–64.2% (Sun et al., 2010; Wu et al., 2011; Wu et al., 2013). Approximately 90% of NSCLC patients harbor exon 19 deletion and exon 21 L858R point mutation. These two types of mutations can be treated by the first-generation EGFR TKIs (such as gefitinib, erlotib, etc.) (Ellis et al., 2015). With the widespread use of the first-generation TKIs, the frequency of house keeper gene EGFR exon 20 mutation (T790M) has been increasingly reported, which results in the acquired resistance to these agents (Lim et al., 2018; Nagano et al., 2018). Amino acid 790 is mutated from Thr to Met, causing the first generation TKI to fail to bind to the ATP site of the EGFR kinase domain. Scientists have developed the second-generation TKIs (such as afatinib, dacomitinib, etc.) (Ramalingam et al., 2014; Soria et al., 2015; Park et al., 2016) and the third-generation TKIs (such as osimertinib) to overcome EGFR T790M-mediated TKI resistance. Osimertinib, as the third-generation EGFR TKI drug, has shown excellent efficacy in animal models and NSCLC patients with EGFR T790M mutation, with an ORR of 70% and a median PFS of 9.9 months (Jänne et al., 2015; Mok et al., 2017). Recent findings indicate that, in patients with stage IB-IIIA EGFR mutation-positive NSCLC, disease-free survival is significantly longer among those who received osimertinib than among those who received placebo (Wu et al., 2020). Overall, the third-generation EGFR TKI possesses high clinical values in both early and late-stage NSCLC patients.

SH-1028 is an irreversible third-generation EGFR TKI, which has been developed by Nanjing Sanhome Pharmaceutical Co., Ltd. with independent intellectual property rights to treat NSCLC. Both SH-1028 and osimertinib are based on a pyrimidine skeleton structure and have a typical third-generation EGFR TKI structure. Compared with osimertinib, SH-1028 is modified on the indole ring, and the original methyl indole is transformed into 6,7,8,9-tetrahydropyrrol [1, 2-a] indole, while retaining its pyrimidine ring. According to the research data of the metabolic pathway of osimertinib, the methyl group in methylindole structure is easily metabolized in the human body ^[37]^. The resulting metabolite (AZ5104) also has a strong inhibitory effect on wild-type EGFR, but may cause rash, diarrhea and other side effects. Since SH-1028 comprises of 6,7,8,9-tetrahydro-pyrrolo [1, 2-a] indol structure, which is more stable and undergoes a unique metabolic pathway, it is possible that the severity of rash and gastrointestinal toxicity may be further reduced.

In the current kinase inhibition and cell proliferation tests, SH-1028 exhibited stronger activity on EGFR sensitive mutations or EGFR T790M resistance mutation than osimertinib, and displayed a good selectivity for EGFR wild-type kinase or cells. Through the phospho-EGFR specific assay and wash-out test *in vitro*, SH-1028 inhibited the phosphorylation of EGFR, and based on its irreversible binding mechanism, there was no reversion of TKI resistance within 24 h after washing out, and the inhibition of EGFR phosphorylation was maintained in both EGFR sensitive mutation and L858R/T790M resistance mutation. Altogether, the findings reveal the mechanism underlying the anti-tumor effect of SH-1028.

For the *in vivo* xenograft model test, animals were administered with 2.5–15 mg/kg/day of SH-1028. The tumor volume in PC-9 (Del 19, EGFR sensitive mutation) or NCI-H1975 (L858R/T790M resistance mutation) xenograft model showed a significant reduction, indicating that SH-1028 could inhibit tumor growth in a dose-dependent manner. In NCI-H1975 xenograft model, the tumor shrinked significantly in 5 mg/kg SH-1028 group, which meant the drug exert anti-tumor effects in low dose. SH-1028 had a high degree of relevance in the efficacy of *in vivo* and *in vitro* models, and it could be speculated that the drug should also play a good anti-tumor effect in clinical patients with such mutation type. Moreover, within this dose range, the exposure of SH-1028 increased with increasing doses, and maintained a good pharmacokinetic/pharmacodynamic relationship with drug efficacy. However, SH-1028 did not show superior efficacy in animals compared to osimertinib, which was inconsistent with the results of *in vitro* activity. There are two main metabolites of osimertinib reported in the literature: AZ5104 and AZ7550 (about 10% of the parent drug) (Cheng et al., 2016; Dickinson et al., 2016; Vishwanathan et al., 2019). AZ5104 is 8-fold more active on EGFR T790M than osimertinib, which is a strong active metabolite. However, this metabolite has a strong inhibitory effect on EGFR wild type, thereby reducing the selectivity of the drug. Although AZ7550 is an active metabolite, its inhibitory effect on EGFR T790M and wild-type mutations is similar or weaker to that of osimertinib (Cross et al., 2014). From the analysis results of metabolites in liver microparticles, it can be seen that SH-1028 may not produce AZ5104-like metabolite during the metabolism of liver microsomes, but can generate metabolites similar to AZ7550. This metabolite is named as Imp3. The results of kinase activity assays showed that Imp3 could exhibit good inhibition efficiency against EGFR-mutated kinases and cells *in vitro*, and had a weaker inhibitory effect on wild-type EGFR, indicating that it can maintain a good selectivity. Through a unique metabolic pathway, SH-1028 may confer fewer adverse reactions, such as diarrhea and rash, compared to osimertinib at the clinical stage. In addition, according to reports (Boire et al., 2020), in patients with terminal lung cancer, the proportion of brain metastases is quite high. Satisfactorily, pharmacokinetic experiments showed that SH-1028 could penetrate the blood-brain barrier, which confirmed it’s greater therapeutic advantages. Now, SH-1028 is in phase I (NCT03603262) and phase II (NCT03823807) clinical trials. All adverse events and efficacy data are in the process of being collected, and a potential improvement in the incidence of diarrhea and rash remains to be confirmed.

## Conclusion

As a novel third-generation EGFR inhibitor, due to its distinct structure and metabolite pathway, SH-1028 exhibits potent activity against EGFR sensitive and resistant (T790M) mutations. Based on its strong inhibitory effect on EGFR sensitive mutations (L858R and Del 19), SH-1028 may exert unique therapeutic properties in future clinical trials and be an alternative option for patients who have developed resistance to the first-generation EGFR TKIs. In sum, it is expected to become the first-line treatment of EGFR mutation-positive advanced NSCLC.

## Data Availability

The raw data supporting the conclusions of this article will be made available by the authors, without undue reservation.

## Abbreviations

NSCLC, non-small cell lung cancer; EGFR, epidermal growth factor receptor; ATP, adenosine triphosphate; TKI, tyrosine kinase inhibitor; IC50, the half-maximal inhibition concentration.
