# Supplementary material for: SH-1028, An Irreversible Third-Generation EGFR TKI, Overcomes T790M-Mediated Resistance in Non-Small Cell Lung Cancer
Source: Front Pharmacol. 2021 Apr 27;12:665253. doi: 10.3389/fphar.2021.665253 (PMC8111447; doi:10.3389/fphar.2021.665253)
Supplement: Supplementary file 1 [file datasheet1.docx]

Supplementary Material

# Supplementary Figures





**Supplementary Figure 1.** (**A**) The tumor growth curve of PC-9 xenograft models after treatment (n = 6). (**B**) The tumor growth curve of A431 xenograft models after treatment (n = 6) (**C**) The body weight of A431 xenograft models after treatment (n = 6). Data were expressed as mean ± SD; *p<0.05, **p<0.01, ***p<0.001 versus Vehicle goup.
